# The isolation and characterization of resident yeasts from the phylloplane of *Arabidopsis thaliana*

**DOI:** 10.1038/srep39403

**Published:** 2016-12-22

**Authors:** Kai Wang, Timo P. Sipilä, Kirk Overmyer

**Affiliations:** 1 Division of Plant Biology, Department of Biosciences, Viikki Plant Science Centre, University of Helsinki, Helsinki, Finland

## Abstract

The genetic model plant *Arabidopsis thaliana* (arabidopsis) has been instrumental to recent advances in our understanding of the molecular function of the plant immune system. However, this work has not yet included plant associated and phytopathogenic yeasts largely due to a lack of yeast species known to interact with arabidopsis. The plant phylloplane is a significant habitat for neutral-residents, plant-growth and health-promoting species, and latent-pathogenic species. However, yeast phylloplane residents of arabidopsis remain underexplored. To address this, resident yeasts from the phyllosphere of wild arabidopsis collected in field conditions have been isolated and characterized. A total of 95 yeast strains representing 23 species in 9 genera were discovered, including potentially psychrophilic and pathogenic strains. Physiological characterization revealed thermotolerance profiles, sensitivity to the arabidopsis phytoalexin camalexin, the production of indolic compounds, and the ability to activate auxin responses *in planta*. These results indicate a rich diversity of yeasts present in the arabidopsis phylloplane and have created culture resources and information useful in the development of model systems for arabidopsis-yeast interactions.

Plants constantly interact with a large number of microorganisms, including bacteria, oomycetes, filamentous fungi, and yeasts. Bacteria are the best studied plant-associated microbes and yeasts have received the least attention. These microbes colonize all plant surfaces, where each plant compartment or structure has its own distinct microbiome. The rhizosphere and endophytic microbiota are the most thoroughly explored and offer numerous examples of microbes modulating plant growth, mineral absorption, and immunity^1^,^2^,^3^,^4^. The phyllosphere is termed as the total surface of aerial parts of plants that is colonized by microorganisms. A variety of bacteria, yeasts and filamentous fungi have been isolated from the phyllosphere of several plant species^5^,^6^,^7^,^8^,^9^,^10^. Physical barriers and multiple chemical factors limit the growth and survival of microbes in the phyllosphere. However, the energy content of leaf surface components and simple sugars leached from the interior tissues^11^,^12^ make the phyllosphere a considerable microbial habitat. Microbes residing in the phyllosphere can have various lifestyles and modes of interaction with the host, being neutral residents, latent pathogens, or plant-health and -growth promoters.

Many plant associated microorganisms, including both growth-promoting and pathogenic microbes, have the ability to synthesize the plant developmental hormone auxin, including the most common auxin, indole-3-acetic acid (IAA) which is responsible for division, elongation and differentiation of plant cells and tissues. Increased attention has been paid to the role of microbial IAA in plant-microbe interactions. It was suggested that auxin production capability might be an important colonization strategy for the phyllosphere environment^13^. IAA-producing bacteria and fungi are able to promote plant growth^14^,^15^. Therefore, they are suggested as potential of bio-fertilizers.

The genetic model plant, *Arabidopsis thaliana* (arabidopsis) is a well characterized model for plant microbe interactions. This encompasses host and non-host pathogen-interaction systems, root-associated microbiome studies, and endophytic fungi. Indeed, arabidopsis has been instrumental in defining the basis of plant immunity, including mechanisms engaged in the perception microbe associated molecular patterns (MAMPs) in MAMP-triggered immunity and *Resistance (R*)-gene function in signalling of effector-triggered immunity^16^,^17^. These studies have focused on fungi, bacteria and oomycetes, but not yeasts. In mammals, a distinct set of immune receptors responsible for perception of yeast-specific MAMPs have been found^18^. Thus, it can be expected that in plants yeasts engage a distinct, and yet unknown, set of immune receptors. The ability of yeast cell wall components to induce plant immunity has been demonstrated^19^. Specifically, baker’s yeast cell-wall and glucopeptide components exhibited elicitor activity in arabidopsis. Knowledge of phyllosphere yeasts and, in general, of plant-yeast interactions is required for a comprehensive view of plant health. However, the power of arabidopsis as a genetic model organism has not been utilized in the study of plant-yeast interactions due to a lack of yeasts known to interact with arabidopsis. In this study arabidopsis phyllosphere resident yeasts were isolated, identified, and physiologically characterized to clarify the nature of their interaction with arabidopsis and create resources for genetic studies of plant-yeast interactions.

## Results

### Isolation of yeasts from the arabidopsis phyllosphere

**Figure 1:**
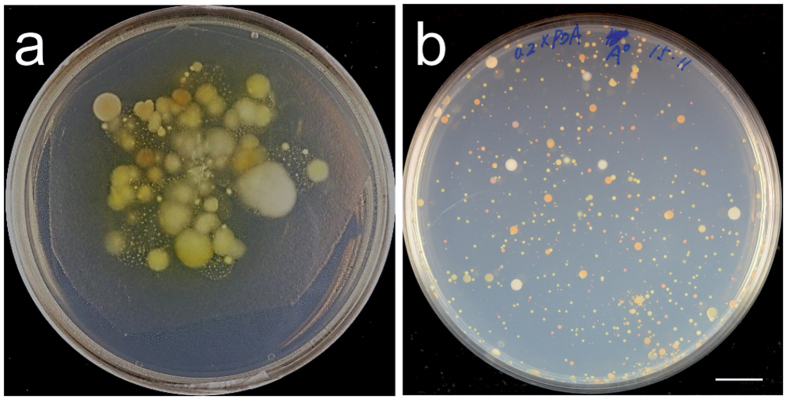
(**a**), The colonies of leaf resident filamentous fungi (5–15 mm in diameter), yeasts and bacteria (0.1–2 mm in diameter) visualized by leaf printing onto a 0.2 × PDA medium plate from a wild *Arabidopsis thaliana*. (**b**), Typical yeast colonies on 0.2 × PDA medium, seven days after plating 20 μL of leaf-wash solution from sample A (Table 1). Similar plates with lower colony density from plating 1:10 or 1:100 dilutions of leaf-wash were used for colony picking. Scale bar: 1 cm.

Few naturally occurring yeasts that are associated with, or pathogenic on, arabidopsis are currently described. To rapidly assess the presence of yeasts, whole rosette imprints of wild arabidopsis collected in the field were cultured using a medium that favours the growth of yeasts. These cultures (Fig. 1a) indicate a high number and apparent wide variety of microbes were present, the majority of which had a colony morphology consistent with yeasts. This result prompted further investigation of arabidopsis associated yeasts.

Wild arabidopsis were collected from three sites in Helsinki; site name, location and samples collected are listed in Table 1. Two sites (Kulosaari and Mustikkamaa) are on islands of the Baltic coast and separated by circa 300 metres, while the third site (Kivikko) was circa 5 km inland. Multiple samples from the same site were always collected from plants at least two metres apart. Serial dilutions of leaf wash solutions were plated and grown at room temperature. A typical plate is depicted in Fig. 1b. Dilute (0.2 x) potato dextrose agar (PDA) was selected as a culture medium with the reasoning that a nutrient-poor, plant-based medium lacking high levels of exogenous amino acids would mimic the conditions in the phyllosphere^11^,^20^. Plates were monitored daily over two weeks and colonies marked and picked as they appeared. Typically 3–5 colonies with similar colour and morphology were picked and streaked on 0.2 x PDA plates to isolate pure single colonies. Isolated colonies were regrown in replicated microtitre plates for storage and DNA isolation. DNA samples were used to PCR amplify rDNA internal transcribed spacer (ITS) sequences for identification. Isolates producing no ITS PCR product were observed by light microscopy to eliminate prokaryotes and PCR was retested with new cultures and DNA samples. Samples failing these tests (60.9% of colonies picked) were eliminated from further analysis. ITS PCR products were subjected to restriction fragment polymorphism analysis to classify the yeasts into operational taxonomic units (OTUs).

For isolate identification and definition of OTUs, restriction patterns and ITS sequencing were used (Supplementary Table S1). For Samples A, B and C collected from Kivikko in May of 2013, there were totally 70 novel yeast strains isolated and identified out of 155 picked colonies. From a total of 56 colonies picked from the November 2013 samples, only one new OTU from Kulosaari and one from Mustikkamaa were found. The low number of new yeasts found in the November samples was partly due to the reappearance of previously isolated OTUs (Table 1 and Supplementary Table S1). However, it was apparent that the diversity of yeasts was much higher in the samples collected in spring than those from autumn. In the M sample from Kulosaari in April 2015, 23 new OTUs were discovered from 32 colonies.

### Identification of yeasts

**Figure 2:**
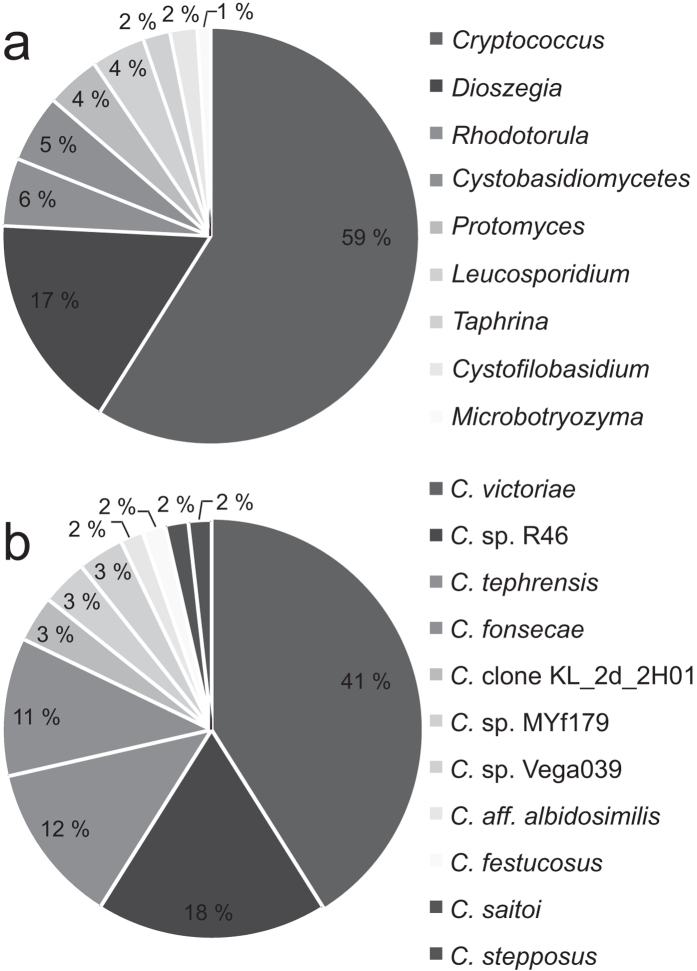
Composition of arabidopsis surface yeasts isolated from the view of genus (**a**) and the species or strain composition of the dominant genus *Cryptococcus* (**b**).

The ITS region from all unique OTUs was sequenced and compared to the NCBI database identifying 95 yeast strains representing 23 species in nine genera (Table 2). There were two species in two genera of the phylum Ascomycota and 21 species in seven genera of the phylum Basidiomycota. Ascomycete yeasts represented 6.3% of total and 93.7% were Basidiomycete. *Cryptococcus* was by far the dominant genus in the arabidopsis phyllosphere, accounting over half of all isolates. OTUs similar to *C. victoriae* strains accounted for 41% of total isolates within the genus *Cryptococcus* (Fig. 2). However, there was a rich diversity of *Cryptococcus* present with a total of 20 OTUs represented.

The behaviour of a selected subset of isolated yeasts was monitored in several physiological assays in order to understand aspects of yeast lifestyle, support the assertion that these species are specifically associated with arabidopsis, and characterize the nature of their host-yeast interaction. The following isolates were selected, as a representative set of genera in this study: *Cryptococcus* sp. OTU 9, *Cryptococcus* sp. OTU 4, *Leucosporidium* sp. OTU 26, *Taphrina* sp. OTU 3, *Protomyces* sp. OTU 1, *Dioszegia* sp. OTU 23 strain 1, *Dioszegia* sp. OTU 23 strain 2, *Cryptococcus* sp. OTU 17, *Cystobasidiomycetes* sp. OTU 32, *Microbotryozyma* sp. OTU 28, *Leucosporidium* sp. OTU 27. Additionally, Baker’s yeast (*Saccharomyces cerevisiae* L40) and fission yeast (*Schizosaccharomyces pombe* FY7519) were used as non-pathogenic control species and the mustard pathogen, *Eremothecium sinecaudum* BSL-1, was used as a known pathogen of *Brassicaceae* family plants^21^.

### Yeast production of indolic compounds

**Figure 3:**
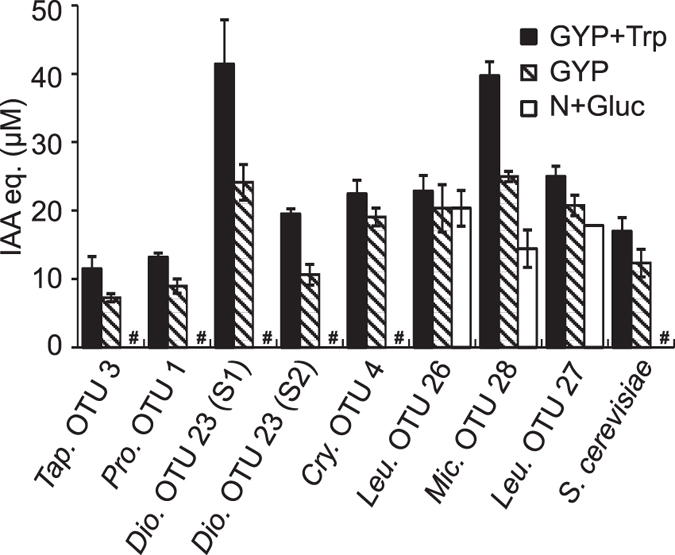
Production of indolic compounds by yeasts. Selected isolates were cultivated in nitrogen base with 1% glucose (N + gluc), glucose-yeast-peptone (GYP) media with and without 0.1% L-tryptophan (Trp). Standard curve was calibrated by using indole acetic acid (IAA) and results are presented as equivalents of IAA (IAA eq.) concentration. ^#^Not detected. Three independent biological replicates were conducted with similar results.

Both pathogenic and growth-promoting microbes are known to produce the plant hormone auxin, prompting examination of this trait in our selected yeasts. Indolic compounds, including indole acetic acid (IAA) and other auxins, were detected using the Salkowski reagent colorimetric assay with supernatants from yeast cultures in media with varied amounts of the auxin precursor tryptophan. Among the 11 selected strains, eight produced indolic compounds when cultivated in glucose yeast peptone (GYP) medium, which contains moderate levels of tryptophan derived from yeast extract and peptone (Fig. 3). Indole compound production, measured in IAA equivalents (IAA eq.), ranged from 7.25 ± 0.60 to 24.98 ± 0.75 μg/ml. When cultivated in GYP supplemented with 0.1% tryptophan, production increased, ranging from 11.48 ± 1.80 to 41.45 ± 6.45. For conditions lacking exogenous tryptophan, a minimal medium of nitrogen base with glucose was used for cultivation. Interestingly, three isolates displayed indole production in minimal medium (Fig. 3), suggesting the ability to produce indoles in the absence of exogenous tryptophan, which better mimics the conditions in the arabidopsis phyllosphere.

### Activation of auxin responses in planta

**Figure 4:**
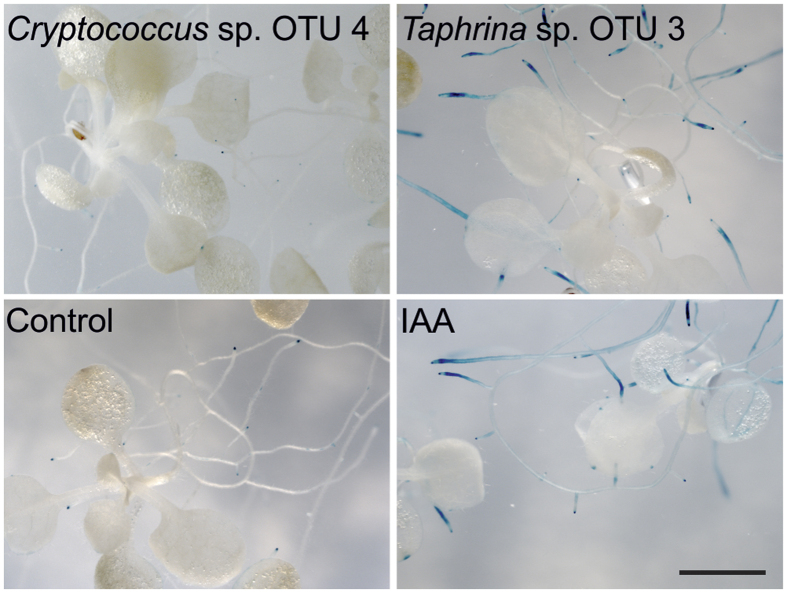
Effect of indolic compounds produced by yeasts on gene expression of auxin regulated genes, monitored as expression of the artificial auxin responsive *DR5* promoter. Two-week-old *in vitro* grown *DR5::GUS* arabidopsis seedlings were treated with the filtered supernatants of five day old yeast cultures for 15 hours. As an example of a positive response, strong GUS expression were detected in seedling roots treated with *Taphrina* sp. OTU 3 supernatant and in the positive control 5 μM IAA treatment. As representative negative results, only light root tip staining was seen with *Cryptococcus* sp. OTU 4 supernatant and the negative control GYP medium treatments. Similar results were observed in three independent biological replicate experiments. Scale bar = 2 mm. See also Supplementary Fig. S1 for results of all treatments.

The artificial auxin-responsive promoter *DR5* is commonly used to monitor the activation of auxin transcriptional response in arabidopsis. To investigate if yeast-derived indolic compounds had auxin activity *in planta*, arabidopsis roots bearing a construct with the *DR5* promoter fused to the β-glucuronidase reporter gene (*DR5::GUS*) in the Col-0 accession were incubated with culture filtrates from five-day-old yeast cultures and monitored by GUS histochemical staining. A 5 μM IAA control treatment and the culture supernatant of *Taphrina* sp. OTU 3, as a representative examples of a positive responses, both exhibit strong blue GUS stains in the roots, indicating induction of DR5-promoter activity (Fig. 4). Similar GUS activity was detected in seedlings treated with culture supernatant of *Taphrina* sp. OTU 3, *Protomyces* sp. OTU 1, *Dioszegia* sp. OTU 23 strain 1, *Saccharomyces cerevisiae, Eremothecium sinecaudum*, as well as light GUS expressions in seedlings treated with *Leucosporidium* sp. OTU 26 and *Leucosporidium* sp. OTU 27 (Supplementary Fig. S1), suggesting the auxin transcriptional response was activated by exogenous auxin from the yeast culture.

Plants treated with an uncultured GYP medium (negative control) or supernatant of *Cryptococcus* sp. OTU 4, are presented as representative examples of negative staining results, which exhibited only light staining of root tips (Fig. 4). Similar negative GUS staining was seen in plants treated with supernatants from *Cryptococcus* sp. OTU 9, *Cryptococcus* sp. OTU 17, *Cystobasidiomycetes* sp. OTU 32, *Dioszegia* sp. OTU 23 strain 2, *Cryptococcus* sp. OTU 4, *Microbotryozyma* sp. OTU 28 and *Schizosaccharomyces pombe* (Supplementary Fig. 1). The indolic compounds produced by strains *Dioszegia sp*. OTU 23 strain 2, *Cryptococcus* sp. OTU 4, and *Microbotryozyma* sp. OTU 28 (Fig. 3) did not trigger auxin transcriptional responses (Supplementary Fig. 1), indicating the presence of indolic compounds with no IAA activity in these species.

### Camalexin sensitivity

**Figure 5:**
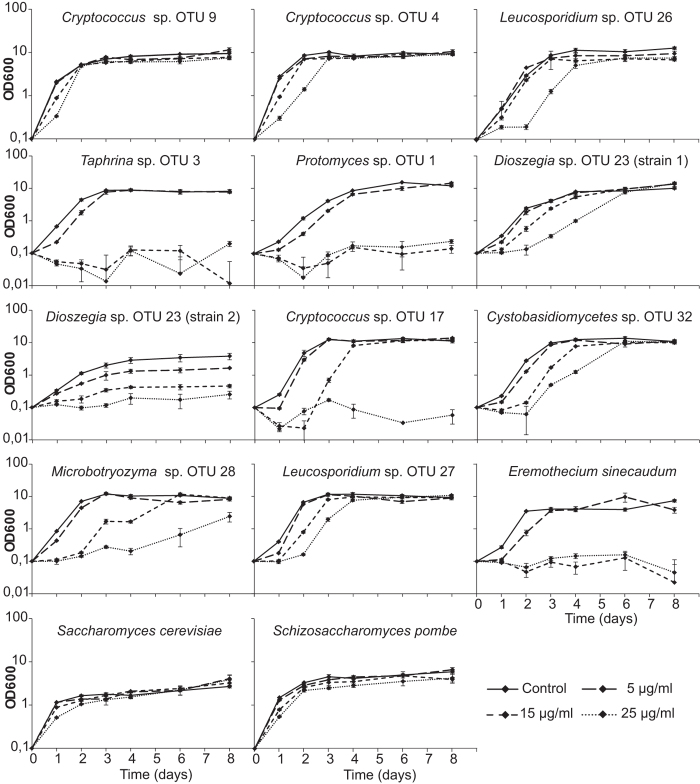
Effect of arabidopsis phytoalexin camalexin on yeast growth. Yeasts were cultured in liquid GYP medium containing 0, 5, 15, 25 μg/ml camalexin, from starting cell density OD = 0.1. Similar results were observed from three independent biological replicate experiments.

We reasoned that pathogenic and non-pathogenic yeasts may differ in their sensitivity to growth inhibition by the arabidopsis antimicrobial phytoalexin, camalexin. Yeasts were cultured in liquid media supplemented with 5, 15 and 25 μg/ml camalexin, revealing high variability in the level of camalexin sensitivity (Fig. 5). Many isolates, including control isolates, exhibited a delay at the initial period of their growth, which suggests these species have inducible camalexin-tolerance mechanisms. Two isolates from genera known to contain plant pathogens (*Taphrina* sp. OTU 3 and *Protomyces* sp. OTU 1) and the control plant pathogen (*Eremothecium sinecaudum*) were sensitive to camalexin at concentrations higher than 15 μg/ml. Isolates from genera not previously known to be phytopathogens were highly resistant to camalexin of the concentration up to 25 μg/ml (Fig. 5).

### Temperature tolerance

**Figure 6:**
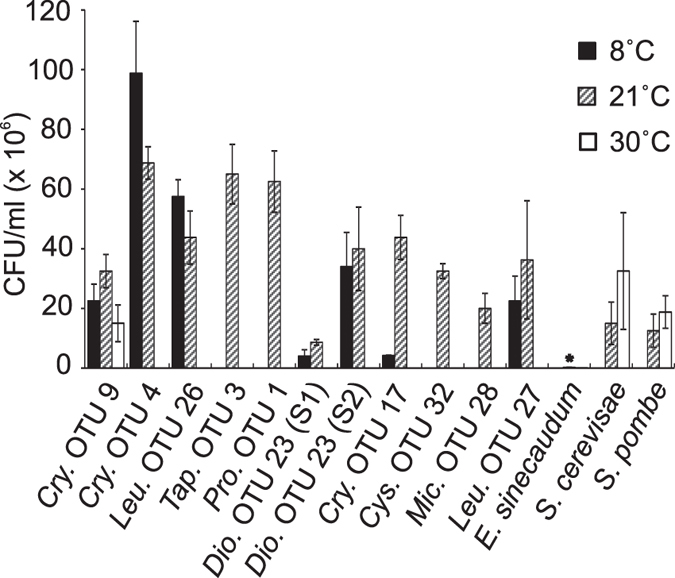
Effect of temperature on yeast growth. Yeasts were first grown on liquid GYP medium for three days and then diluted to OD = 1. Serial of dilutions (OD = 10^−2^, 10^−3^, 10^−4^, 10^−5^, 10^−6^, 10^−7^) were plated on GYP agar medium for grown at different temperature conditions (8 °C, 21 °C, 30 °C, 37 °C). Colony forming units (CFUs) were counted to quantify growth after seven days. No isolate grew at 37 °C. Similar results were observed from three independent biological replicate experiments.

Most yeasts are mesophilic and grow optimally at temperatures between 20 and 25 °C, but still many species can grow between 2 and 10 °C. All the strains in this study were isolated at 21 °C from plants growing at low temperatures. In order to characterize the thermotolerance profiles and identify cold-adapted yeasts, growth under four temperature conditions (8, 21, 30, 37 °C) was tested. Seven out of 11 selected strains grew at 8 °C, indicating a high number of cold-adapted yeasts among our isolates. OTU 4 and OTU 26 grew better at low temperature than at room temperature. Only one strain (OTU 9) grew at 30 °C and at 37 °C (Fig. 6).

## Discussion

In this study we have isolated yeasts from the arabidopsis phylloplane. Among the yeast isolates presented here, 22 OTUs belong to the phylum Basidiomycota, which was much more abundant than the phylum Ascomycota with only two OTUs. The Ascomycete and Basidiomycete ratio is less than those of yeasts isolated in tropical regions^5^,^7^,^22^, subtropical areas^8^ and temperate areas^23^,^24^. However, Ascomycete yeasts may also dominate in the phyllosphere of plants in tropical regions, when the isolation was performed using a higher temperature^7^. Interestingly, a study of seasonal dynamics demonstrated the abundance of *Ascomycete*s increased gradually from spring to autumn^23^.

In Finland and other northern counties, arabidopsis seeds germinate in the autumn and grow until usually November, when temperatures drop, overwintering as a small rosette, often under snow cover, until growth resumes in the spring, typically in April. The lifecycle is then completed with seed set in late May or early June. Accordingly, a high percentage of low-temperature adapted yeasts were isolated from plants sampled in May, whose rosettes had overwintered under snow. Some plant pathogens are specialized for infection during winter and there is currently no genetically tractable model pathosystem for such interactions. Further studies are required, however, some of the isolates presented here may have potential for the development of a low temperature adapted pathogen model system.

Auxin production by a plant-associated microorganism was first reported by Kaper and Veldstra^25^. Auxin of microbial origin has been shown to play a wide variety of roles^26^. Within and between microbes, IAA can function as a signalling molecule regulating microbial gene expression, promoting growth-form switching, and potentially as a quorum sensing molecule^26^,^27^,^28^.

In pathogenic microbes, IAA functions in tumour or gall formation, suppression of plant immune-signalling and aiding pathogen ingress via induction of plant cell wall expansion and weakening^29^. Remarkably, two potentially pathogenic OTUs isolated here (OTU 1 and 3), both of which exhibited the ability to produce indolic compounds and induce an auxin response *in planta*, both come from genera known to induce plant tumours as disease symptoms (*Protomyces* and *Taphrina*). In plant-growth promoting microorganisms, IAA results in enhanced root-proliferation, increased mineral adsorption, and increased basal defence^9^,^14^. Given the variety of these responses, diverse model systems are needed for the study IAA in plant-microbe interactions in different contexts. Although IAA-producing yeasts have been characterized from many other plants, and IAA-producing bacteria and fungi that interact with arabidopsis are known, this study offers the first opportunity for a yeast-arabidopsis interaction involving this plant hormone. In this study, we isolated several potentially arabidopsis-associated yeasts with the ability to produce indolic compounds. Among the tested eleven yeast isolates, eight isolates exhibited significant production of indolic compounds when cultivated in various media with different levels of exogenous tryptophan. Tryptophan is the main precursor for IAA biosynthesis pathway in most organisms and there are five different biosynthesis pathways known leading from tryptophan to IAA^26^. Additionally, a tryptophan-independent pathway exists in bacteria and fungi^27^,^30^. Three isolates (*Leucosporidium* sp. OTU 26, *Microbotryozyma* sp. OTU 28 and *Leucosporidium* sp. OTU 27) exhibited the production of indolic compounds in the absence of tryptophan in the growth medium. Culture filtrates from two of these isolates (OTU 26 and OTU 27) had *in planta* auxin activity (Supplementary Fig. S1). Most studies reporting microbial auxin production utilize growth media containing exogenous tryptophan or containing yeast extract or peptone, which contain high levels of tryptophan. In contrast, tryptophan was reported as a low abundance amino acid in oat and barley leaf exudates^20^. Here we demonstrate yeasts with the ability to produce auxins or other indolic compounds under conditions more similar to the low tryptophan levels expected in the phylloplane. These yeasts may use either endogenous *de novo* tryptophan biosynthesis or a non-tryptophan-dependent IAA/indole biosynthesis pathway.

In general, most of the assayed yeast-culture filtrates that contained indolic compounds were also shown to have auxin activity in the induction of a transcriptional marker in arabidopsis suggesting that they contain auxins. It has been proposed that indolic compound production by yeasts is a general adaptation strategy for plant phyllosphere environment^9^. Since our yeast strains were isolated from wild arabidopsis plants, without any disease symptoms, indolic compound production may be an important indicator for plant associated beneficial or neutral microbes.

The indole alkaloid, camalexin (3-thiazol-2′-yl-indole), is the major anti-microbial phytoalexin in arabidopsis. Like other low molecular weight antimicrobial compounds, camalexin synthesis is rapidly induced by plant pathogens^31^,^32^,^33^,^34^. Several arabidopsis pathogens are known to be sensitive to camalexin^35^, which contributes to arabidopsis pathogen immunity^36^,^37^. Cell membrane disruption is considered as the primary mode of camalexin toxicity against bacterial, fungal and plant cells^38^. Sensitivity to camalexin is not restricted to strictly to pathogenic species; both resistant and sensitive isolates were found within *Botrytis cinerea*^36^. However, fungal pathogens generally have a much lower toxicity threshold than bacterial pathogens and host plant cells^38^. In this study, the high sensitivity of three potentially pathogenic yeast isolates (Fig. 5) confirmed the low toxicity threshold of pathogenic fungi. It has been shown that culture media supplemented with 20 μg/ml camalexin caused a significant decrease in spore germination and hyphal growth of *Botrytis cinerea* isolates^36^, which is in accordance to the sensitivity of three potentially pathogenic yeast isolates to 15 μg/ml camalexin.

Several fungi are reported to have tolerance against camalexin by its active degradation into less toxic compounds^39^,^40^. Similarly, the non-pathogenic yeast strains exhibited high tolerance in cultures with camalexin concentrations up to 25 μg/ml. Although they might be sensitive higher camalexin concentrations^38^, these are not realistic to the situation in the phyllosphere of arabidopsis, where it has been estimated that camalexin accumulation reached a maximum of 8 μg/g leaf fresh weight in arabidopsis after inoculation with a bacterial pathogen^41^.

Remarkably, in the control experiments, higher camalexin tolerance was a characteristic of the non-pathogenic species (*S. pombe* and *S. cerevisiae*), while the control pathogen (*Eremothecium*) was more sensitive. This trend also held among the strains isolated here, genera previously known to be plant pathogens were sensitive, while genera with no previous report of plant pathogenicity tended to be tolerant (see Fig. 5 and discussion of the individual genera below). This is consistent with the pathogen lifestyle and virulence strategy; pathogens do not require camalexin tolerance as they can avoid camalexin, via subverting immune signalling pathways with effector proteins and other virulence mechanisms. Thus we contend that, although not a perfect indicator all of the time, camalexin sensitivity can be used as a guide in the identification of potential latent pathogens among phylloplane resident microbes.

All the yeast genera isolated from *the* arabidopsis phyllosphere have been previously demonstrated to be associated with plants. Several have also been previously found to be associated with arabidopsis. Recently, Alger *et al*.^42^ characterized the arabidopsis phyllosphere using metagenomics. This study independently verified the presence of OTUs in the genera *Protomyces, Dioszegia, Leucosporidium*, and *Rhodotorula*, in the phyllosphere of arabidopsis collected from two distant sites in Germany. This suggests that these yeasts may indeed belong to the recurring core phyllosphere of arabidopsis.

The characteristics of each genus found in our isolations are individually discussed below.

*Cryptococcus* is the dominant genus in phyllosphere of arabidopsis in this study. Members of the genus *Cryptococcus* are widely distributed and were described as a dominant species in the phyllosphere of other plants, such as carnivorous plant^9^, spruce and birch^23^. Another group of yeast commonly found in the phyllosphere of many plants, *Dioszegia* spp. have been previously detected on plant leaves and roots, and even polar desert soil^10^,^43^,^44^,^45^. Some novel psychrophilic species in this genus isolated from Antarctica have been previously described^45^. The world-wide distribution of the *Cryptococcus* and *Dioszegia* species may due to the extreme temperature tolerance and the ability to utilize the nutrients from harsh environments. Among 11 tested isolates, two *Dioszegia* isolates and two *Cryptococcus* isolates performed active growth at low temperature, suggesting the possible cold adaptation in northern climates. A *Dioszegia* sp. was previously found in the arabidopsis phyllosphere^42^ and found to act as a so called “hub” microbe, which are species that have a role in organizing the microbiome community. Specifically, presumably via direct microbe-microbe interactions, *Dioszegia* spp. shaped the prokaryotic microbiome of the plants on which they resided^42^. Due to the importance of these species, we have sequenced the genome of the *Dioszegia* sp. OTU 23 isolated in this study. Analysis of this genome will be presented elsewhere.

The genus *Rhodotorula* is a group of pigmented yeasts, widely variant in colour, being cream to orange, red, pink or yellow. As a common environmental microbe, *Rhodotorula* species have been cultured from water, milk, fruit juice, soil and even air samples^46^. Interestingly, *R. glacialis* has been studied as an oleaginous yeast, which might be used for the production of single cell oils^47^. The studies also demonstrated that under appropriate conditions, this yeast accumulates high amount of lipids. Additionally, siderophores produced by *Rhodotorula* strains exhibited antifungal activity against plant pathogens including *Botrytis cinerea*^48^. *Holtermannia* is a group of yeast with wide-spread distribution. *Holtermannia* strains have been isolated from many natural and artificial substrates from different parts of the world^49^.

Two of the potentially pathogenic yeasts isolated are from the genera, *Taphrina* and *Protomyces*, which both belong to the *Taphrinomycotina*. Members of this early diverging *Ascomycete* subphylum are considered to be ancient lineages and as such are important for understanding fungal evolution and the evolution of pathogenicity. Established by Unger in 1832, the genus *Protomyces* was described as pathogens that cause galls on stems, leaves or fruits on host plants in the families *Compositae* and *Umbelliferae. Protomyces* has been strictly defined based morphologically on cell sizes and on their host range, where only yeasts pathogenic on *Compositae* and *Umbelliferae* belong to *Protomyces*^50^. This is among the first reports to isolate or detect a *Protomyces* sp., as defined by ITS sequences, from a plant outside of these two host families. ITS sequences of the *Protomyces* spp. described here (OTU 1 and OTU 2), place them firmly within the *Protomyces*. However, these ITS sequences are only 97% and 95% similar to that of their closest BLAST hit *Protomyces inouyei*. This suggests that OTU 1 and 2 are novel *Protomyces* species with hosts outside of the defined range. Many yeasts morphologically similar to *Protomyces* have been characterized but excluded from the genus based on their host plants^50^; however, descriptions of these species did not include molecular data (ITS sequences). Taken together, these data suggest that the definition of the genus *Protomyces* should be revised. It has been previously noted that genus and species demarcation in *Protomyces* and the related genera, *Burenia, Protomycopsis, Taphridium*, and *Volkartia,* are unclear and likely incorrect and that extensive molecular comparisons are needed^51^. Relationships within *Protomyces* are currently being addressed by sequencing the genome of OTU 1 and several reference *Protomyces* species (Wang, Sipilä and Overmyer, unpublished data). This work will be presented elsewhere.

As a genus closely related to *Protomyces, Taphrina* contains nearly 100 described species that are parasitic on different families of primarily woody plants^52^. Some *Taphrina* species cause diseases on fruit trees, of which the symptoms are diverse malformations of leaves and other infected tissue^53^.

Species of genus *Leucosporidium* were originally transferred from *Rhodotorula*^54^. Most of the species of this genus were isolated from cold climates^51^, some of which were described for the ability to degrade phenol and phenol-related compounds^55^. One *Leucosporidium* isolated here is suggested to be cold-adapted because of its active growth at low temperature. The capacity for biodegradation of phenol and phenol-related compounds by *Leucosporidium* isolates found in this study may be of biotechnological interest and warrants further study. The genus *Leucosporidium* is phylogenetically closely related to *Leucosporidiella*. One of the isolates discovered in this genus (OTU 27), which was most closely related to *Leucosporidium golubevii* isolated from river water in Portugal^54^, grew actively at low temperature in accordance with the psychrophilic nature of this genus. In addition, an unknown isolate was collected with 86% ITS identity to *Microbotryozyma collariae*, which was described recently as a novel species^56^.

The yeast species in *Cystofilobasidium* are widely distributed mainly in cold climates. Interestingly, a *C. capitatum* strain (PPY-1) was isolated from soil and was suggested may produce novel enzymes that can degrade pectin at low temperature^57^. Subsequently, a cold-active extracellular pectin lyase from the strain PPY-1 has been purified^58^. The ability to produce plant cell wall degrading enzymes may be a survival strategy for several microbe groups. Production of pectin lyase and related enzymes may be relevant to our strains, future testing for enzymes with these characteristics may have practical application.

Metagenomics is currently the state of the art for the study of plant microbiota and application of this technique to explore the fungi and yeasts in the arabidopsis phyllosphere microbiome gives a more comprehensive picture of the species present^42^ and is complementary to this study. The purpose of the present study was to make an initial survey of yeast in the arabidopsis phyllosphere, isolate strains for use in future studies and physiologically characterize these strains to aid in selecting appropriate strains for developing new model systems with arabidopsis. Currently, the majority of work on molecular plant-microbe interactions with arabidopsis utilize only a small number of pathogenic species, including mostly bacteria and fungi^59^,^60^, but no yeasts. There is a need to diversify the study of plant pathology and include molecular work on plant yeast interactions. The strain resources and information generated in this study will contribute significantly to this effort.

## Methods

### Sample collection and yeast isolation

Healthy rosettes of arabidopsis (*Arabidopsis thaliana)* were collected from three distinct sites (in the Kivikko, Kulosaari and Mustikkamaa districts) in Helsinki, Finland (Table 1). The month average temperature of Helsinki in April, May and November are 4 °C, 10 °C and 0 °C (Finnish Meteorological Institute; http://en.ilmatieteenlaitos.fi). Plants were collected by sterile scissor and forceps and were placed in sterile 50 ml centrifuge tubes. Samples were kept at cold-room (4 °C) until yeast isolation procedures. As a pre-wash to remove surface dust, the leaves were vortexed two times in a 2 ml tube containing 1 ml sterile water for 3 seconds. Leaves were transferred into another 2 ml tube containing 1 ml MQ water with 0.025% Silwet-L77 and were shaken gently for 4 hours on a rocking platform. Serial dilutions of leaf wash solution were plated onto 0.2 × PDA (potato dextrose agar, Sigma-Aldrich) medium. Single colonies were picked and were streaked twice on 0.2 × PDA medium with 100 μg/ml Ampicillin for purification. Long-term storage of wash solutions as well as purified strains was performed in 50% sterile glycerol at −80 °C.

### DNA extraction

Yeast were cultivated in 2 ml 0.2 × PDB (Potato dextrose broth, Sigma-Aldrich) for 5 days. Cells were pelleted by centrifugation for 5 min at 12,000 *g* and the pellet was suspended in 200 μl lysis buffer (100 mM Tris-HCl pH8.0, 50 mM EDTA, 500 mM NaCl). Glass beads (0.3 g) and 200 μl phenol/chloroform/isoamyl alcohol were added and vortexed at high speed for 3 min. Cells were briefly vortexed after adding 200 μl TE buffer. The samples were centrifuged 5 min at maximum speed. The aqueous layer was transferred to a clean 2 ml centrifuge tube, ethanol precipitated, pelleted by centrifugation and resuspended in 0.4 ml TE buffer. Following DNase-free RNase A treatment (30 μl of 1 mg/ml, 5 min at 37 °C) samples were ethanol precipitated as above and resuspended in 100 μl TE buffer.

### Yeast identification

Sequences of the ITS (internal transcribed spacer) region were determined by polymerase chain reaction (PCR) products from yeast genomic DNA. PCR products were obtained using forward primer ITS3 (5′-CTTGGTCATTTAGAGGAAGTAA-3′) and reverse primer ITS4 (5′-TCCTCCGCTTATTGATATGC-3′). Each reaction mixture contained 2 μl 10 × fire polymerase buffer B (Solis BioDyne), 1.6 μl MgCl_2_ (25 mM), 0.4 μl dNTP, 0.4 μl of each primer (10 mM). After denaturation of DNA at 94 °C for 5 minutes, 35 cycles of amplification with the following thermocycling program: denaturation at 94 °C for 20 s, followed by 10 s at 50 °C for primer annealing and 30 s at 72 °C for extension. Final extension 5 minutes at 72 °C was used. The PCR products were observed by separating the fragments with 1.5% agarose gel electrophoresis. ExoSAP (Thermo Scientific™ Exonuclease I, Shrimp Alkaline Phosphatase) treatment of individual PCR products was performed to remove residual primers.

Restriction enzymes HaeIII and TaqI (Thermo scientific) were chosen for ITS fragment digestion, following the manufacturer’s instructions and fragments were observed by separation by electrophoresis using a 3% agarose gel. Isolates were classified as OTUs (operational taxonomic units) on the basis of a unique ITS restriction pattern with the enzymes HaeIII and TaqI and corresponding ITS amplicon length. Representatives of each OTU were selected for sequencing with ITS4 and ITS1 (GGAAGTAAAAGTCGTAACAAGG) primers. The complete ITS region was assembled from the sequencing results (Chromas Lite; Ape). ITS comparison to described fungi species was performed using BLAST (Basic Local Alignment Search Tool) at the NCBI (National Centre for Biotechnology Information) database.

### Indolic compound production and GUS staining

The production of indolic compounds of selected strains was monitored using Salkowski reagent^61^. Three different media (GYP, GYP with 0.1% L-tryptophan, and nitrogen base with 1% glucose) were used. Yeasts were cultured in liquid medium in 96 deep-well plate (P8116, Sigma-Aldrich) for three days from starting cell density OD = 0.1. Breathable film (A9224, Sigma-Aldrich) was used for sealing the plates. One ml yeast cells were centrifuged at 12,000 g for 5 min, and 0.5 ml supernatant was mixed well with 0.5 ml Salkowski regent (12 g of FeCl_3_ per litre in 7.9 M H_2_SO_4_). The mixture was kept at darkness for 30 min. The red colour development was quantified spectrophotometrically (Agilent 8453) as absorbance at wavelength 530 nm. A calibration curve was established using indole-3-acetic acid (IAA) with concentrations of 1, 2, 4, 5, 6, 8, 10 μg/ml. Experiments were repeated independently three times. An arabidopsis *DR5::GUS* transgenic line in the Col-0 accession (obtained from the Nottingham Arabidopsis Stock Centre; NASC; http://arabidopsis.info) was cultivated in 0.5 × Murashige and Skoog^15^ (M5519, Sigma, USA) with addition of 1% (w/v) agar and 1% (w/v) sucrose (pH = 5.7). Seeds were surface sterilized by washing with 70% ethanol with 2% Triton X-100 for 5 min, and washed two times with absolute ethanol. Seedlings were grown in 6 well cell culture plates (CLS3516, Corning Costar), with 10 seeds in each well. Plants were placed in growth chamber (16/8, 24 °C). Ten-day-old *DR5::GUS* seedlings were treated with 1 ml filtered supernatants of 5 day yeast cultures (GYP with 0.1% L-tryptophan) overnight. Positive control group was treated with 1 ml 5 μM IAA, and 1 ml medium for negative control group. For histochemical staining, seedlings were fixed with ice-cold 90% acetone for 1 h, washed three times with ice-cold wash solution (36 mM Na_2_HPO_4_, 14 mM NaH_2_PO_4_, pH 7.2), 30 min for each wash. Seedlings were vacuum infiltrated and kept at room temperature in GUS staining solution (1 mM 5-bromo-4-chloro-3-indolyl b-D-glucuronide dissolved in methanol, 5 mM potassium ferricyanide and 5 mM potassium ferrocyanide in 50 mM sodium phosphate buffer, pH 7.2). Stained seedlings were washed three times with absolute ethanol and were kept in 70% ethanol. Experiments were repeated independently three times.

### Camalexin and temperature tolerance assays

Eleven selected strains and three control strains were cultivated in liquid GYP medium in 96 deep-well plate, containing 0, 5, 15, 25 μg/ml camalexin (SML1016, Sigma-Aldrich). The mustard pathogen, *Eremothecium sinecaudum* BSL-1 (CBS 8199) used as a control was obtained from CBS-KNAW (http://www.cbs.knaw.nl). Plates were shaken at 850 rpm at 21 °C in a growth chamber. Absorbance density at 600 nm of cultures were measured at several time points (day 0, 1, 2, 3, 4, 6, 8) as indication of yeast density. Yeast growth at different camalexin concentrations were measured from starting cell density OD = 0.1. Experiments were repeated independently three times. For temperature growth experiments, yeast strains were first cultivated in liquid GYP medium for three days, then diluted to OD = 1 with medium. Serial dilutions (10^−1^ to 10^−7^) were prepared. Drop plating with 2 μl of each dilution was conducted in square GYP agar plates. The strains were cultivated at 8 °C, 21 °C, 30 °C and 37 °C conditions. Colony forming units (CFUs) were counted to determine growth after seven days. Experiments were repeated independently three times.

## Additional Information

**How to cite this article**: Wang, K. *et al*. The isolation and characterization of resident yeasts from the phylloplane of *Arabidopsis thaliana. Sci. Rep.* **6**, 39403; doi: 10.1038/srep39403 (2016).

**Publisher's note:** Springer Nature remains neutral with regard to jurisdictional claims in published maps and institutional affiliations.

## Supplementary Material

Supplementary Information

## Figures and Tables

**Table 1:** Leaf samples collected from three different sites in Helsinki.

Date	Collection site^a^	GPS coordinates^b^	Sample	Colonies picked	Yeast strains
May 2013	Kivikko	Lat: 60.23270 Lon: 25.06191	A	47	13
			B	51	41
			C	57	16
Dec 2013	Kulosaari	Lat: 60.18376 Lon: 24.99241	F	18	1
			F’	10	0
	Mustikkamaa	Lat: 60.18070 Lon: 24.99463	G	28	1
			H	nd^c^	nd
Apr 2015	Kulosaari	Lat: 60.18376 Lon: 24.99241	M	32	23
			N	nd	nd

^a^Collection sites are all districts in Helsinki, Finland.

^b^Lat: latitude; Lon: longitude.

^c^nd: no data.

**Table 2:** Yeast OTUs isolated from the wild arabidopsis phyllosphere.

OTUs^a^	Closest species^b^ (strain/isolate)	No.^c^	ITS len^d^	Sub^e^	ITS sim^f^	Gaps	Acc no.^g^
**Ascomycota**							
Taphrinomycetes							
1	*Protomyces inouyei* JCM 22201	3(3)	462	19/469	96%	10/469	LT602858
2	*Protomyces inouyei* JCM 22201	1(1)	463	4/470	95%	11/470	LT602859
3	*Taphrina carnea* strain CBS 332.55	2(2)	578	4/579	99%	1/579	LT602860
**Basidiomycota**							
Tremellomycetes							
4	*Cryptococcus* sp. R46	4(3)	458	1/458	99%	1/458	LT602861
5	*Cryptococcus festucosus* CBS 11757	1(1)	474	2/474	99%	2/474	LT602862
6	*Cryptococcus fonsecae* DSM 26992	2(2)	457	1/457	99%	0/457	LT602863
7	*Cryptococcus* sp. MYf180	1(1)	439	1/439	99%	1/439	LT602864
8	*Cryptococcus fonsecae* KF921	4(4)	453	1/453	99%	0/453	LT602865
9	*Cryptococcus aff. albidosimilis* GW_OTU40	1(1)	538	1/539	99%	1/539	LT602866
10	*Cryptococcus saitoi* P01D003	1(1)	561	2/563	99%	2/563	LT602867
11	*Cryptococcus tephrensis*	6(3)	435	0/435	100%	0/435	LT602868
12	*Cryptococcus stepposus* P25B002	1(1)	568	2/570	99%	2/570	LT602869
13	*Cryptococcus tephrensis*	1(1)	450	58/455	87%	7/455	LT602870
14	*Cryptococcus* clone KL_2d_2H01	2(2)	409	0/409	100%	0/409	LT602871
15	*Cryptococcus victoriae* HB 1221	11(1)	447	1/448	99%	1/448	LT602872
16	*Cryptococcus* sp. MYf179	1(1)	421	8/421	98%	0/405	LT602873
17	*Cryptococcus victoriae* M5-10C-5	9(5)	432	0/432	100%	0/432	LT602874
18	*Cryptococcus victoriae* M5-10C-5	1(1)	431	1/431	99%	0/431	LT602875
19	*Cryptococcus* sp. Vega039	2(1)	432	1/432	99%	0/432	LT602876
20	*Cryptococcus victoriae* M5-10C-5	1(1)	432	2/432	99%	0/432	LT602877
21	*Cryptococcus victoriae* HB 1221	1(1)	431	0/431	100%	0/431	LT602878
22	*Cystofilobasidium capitatum* ATCC 24507	2(1)	518	0/518	100%	0/518	LT602879
23	*Dioszegia crocea* DBVPG 5030	16(7)	366	0/366	100%	0/366	LT602880
24	*Cryptococcus* sp. R46	3(3)	512	0/513	99%	1/513	LT602881
25	*Cryptococcus* sp. R46	3(1)	475	4/476	99%	1/476	LT602882
Microbotryomycetes							
26	*Leucosporidium creatinivorum* JCM10699	3(2)	511	4/511	99%	4/511	LT602883
27	*Leucosporidium golubevii* PYCC 5759T	1(1)	523	1/524	99%	1/524	LT602884
Urediniomycetes							
28	*Microbotryozyma collariae* ATCC MYA-4666	1(1)	528	3/528	86%	28/528	LT602885
29	*Rhodotorula glacialis* AB23-2	2(2)	523	2/524	99%	1/524	LT602886
30	*Rhodotorula* sp. YSAR13	1(1)	514	9/514	98%	0/514	LT602887
31	*Rhodotorula sonckii*	2(2)	472	60/446	87%	16/446	LT602888
Cystobasidiomycetes							
32	*Cystobasidiomycetes* sp. KSS-2008 5-19	2(2)	563	34/568	94%	10/568	LT602889
33	*Cystobasidiomycetes* sp. DSM 28479	3(3)	538	1/537	99%	0/537	LT602890

^a^Operational taxonomic units (OTUs) identified in this study.

^b^Closest known related species as determined by best BLAST hit with ITS sequences; results represent searches from NCBI databases in June 2016.

^c^No.: Number of isolates (numbers of isolates sequenced).

^d^ITS len: ITS length (bp).

^e^Sub: Substitution.

^f^ITS sim: ITS similarity.

^g^ITS sequences have been deposited in European Nucleotide Archive under the listed accession numbers (Acc no.).
