# Supplementary material for: The isolation and characterization of resident yeasts from the phylloplane of Arabidopsis thaliana
Source: Sci Rep. 2016 Dec 22;6:39403. doi: 10.1038/srep39403 (PMC5177952; doi:10.1038/srep39403)
Supplement: Supplementary Information [file srep39403-s1.pdf]

Supplementary Information

The isolation and characterization of resident yeasts from the *Arabidopsis* phylloplane.

Kai Wang, Timo P. Sipilä and Kirk Overmyer*

Division of Plant Biology, Department of Biosciences, Viikki Plant Science Centre, University of Helsinki, Helsinki, Finland.

*Corresponding Author: Kirk Overmyer (kirk.overmyer@helsinki.fi)

Supplementary Table S1. Isolates within the same OTU. List of all isolates according to the OUTs to which they belong. The rDNA Internal transcribed spacer (ITS) PCR product and its digestion patterns with the restriction endonucleases Hae III and Taq I were used to define the OTUs and are also listed.

OTUs	Isolates	Digestion patterns (Hae III)	Digestion patterns (Taq I)
1	C29, C2-11, C2-15	800,500,250,	nd
2	A14	600,500,350,50,	nd
3	M12, M11	[a] nd	nd
4	B32, B33, B2-6, B34	375,100,50,25	200, 175, 50
5	M9	nd	nd
6	M15, M16	nd	nd
7	C68	350,200,	nd
8	M4, M5, M6, M7	nd	nd
9	M1	nd	nd
10	G2-20	nd	nd
11	B36, B39, A63, A64, A65, A66	400,150,	225, 200, 50
12	B35	550	250, 50
13	B24,	350,75,	nd
14	C69, C70	350,150,	nd
15	B37, B38, B47, B48, B49, B50, B51, B56, B57, B58, B59	350,200,	nd
16	M3	nd	nd
17	C71, M18, ,M19, M10, M8, C72, C73, C74, C75	350,200,	nd
18	M2	nd	nd
19	B84, B87	350,200,50	nd
20	M20	nd	nd
21	B2-5,	nd	nd
22	B88, B90	400,150,100	nd
23	A64, B40, B43, B2-3, B2-4, A4, B30, A3, A5, A6, B41, B42, B52, B53, B54, B55	250,200,50,	225, 200, 50
24	C2-7, C2-9, F2-23,	nd	nd
25	B45, B44, B46	550,350,100,50,	200, 175, 50
26	B85, B2-2, B86	550,125,	nd
27	C2-14	nd	nd
28	C2-13	nd	nd
29	A20, A21	550,125,	nd
30	A9	550,50,	nd
31	M14, C2-12	nd	nd
32	M22, M24	nd	nd
33	M17, M21, M23	nd	nd

[a] nd: no data.

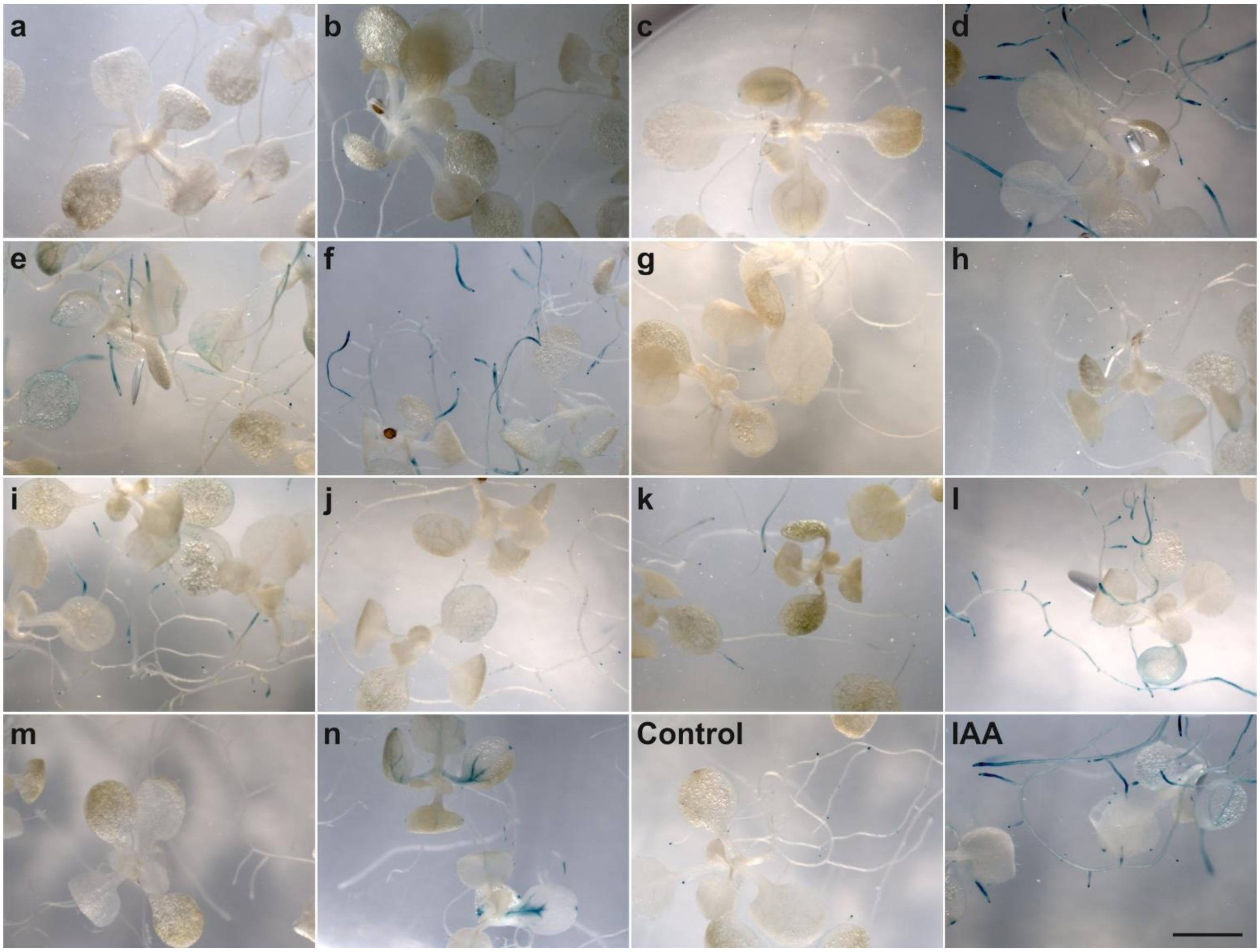

Supplementary Figure S1. **Activation of plant auxin response by yeast culture filtrates.** Activation of the artificial auxin responsive (DR5) promoter was monitored by GUS histochemical staining in DR5::GUS *Arabidopsis* grown in ½ MS agar medium with 1% sucrose (pH=5.7). 2-week-old DR5::GUS *Arabidopsis* seedlings were treated with the filtered supernatants of 5-d yeast cultures for 15 hours. No GUS expression was detected in seedling treated with supernatant of (a) (*Cryptococcus* sp. OTU 9), (b) (*Cryptococcus* sp. OTU 17), (c) (*Cystobasidiomycetes* sp. OTU 32), (g) (*Dioszegia* sp. OTU 23 (strain 2)), (h) (*Cryptococcus* sp. OTU 4), (j) (*Microbotryozyma* sp. OTU 28), (m) (*Schizosaccharomyces pombe*) and GYP medium with 0.1% tryptophan. Strong GUS expressions were detected in seedling treated with (d) (*Taphrina* sp. OTU 3), (e) (*Protomyces* sp. OTU 1), (f) (*Dioszegia* sp. OTU 23 (strain 1)), (l) (*Saccharomyces cerevisiae*), (n) (*Eremothecium sinecaudum*) and IAA (5 μM). Light GUS expressions were detected in seedlings treated with (i) (*Leucosporidiella* sp. OTU 26) and (k) (*Leucosporidium* sp. OTU 27). Similar results were observed from other two duplicate experiments. Scale bar = 2 mm.
